# Risk-of-Bias Assessment of Target Trial Emulation Studies at Regulatory and Policy Decision Points Along the Medical Product Life Cycle: Protocol for a Scoping Review and Stakeholder Survey

**DOI:** 10.2196/107499

**Published:** 2026-10-27

**Authors:** Anni Kokolahti, Saara Puustinen, Martin Russek, Christoph Röthlein, Cornelia Becker, Jonas Peltner, Ines Costa, Sirpa Hartikainen, Britta Haenisch, Cláudia Furtado, Anna-Maija Tolppanen, Christina Tikka

**Affiliations:** 1 School of Pharmacy University of Eastern Finland Kuopio Finland; 2 Federal Institute for Drugs and Medical Devices (BfArM) Bonn Germany; 3 Portuguese National Authority of Medicines and Health Products (Infarmed) Lisbon Portugal; 4 German Center for Neurodegenerative Diseases Bonn Germany; 5 Center for Translational Medicine University of Bonn Bonn Germany; 6 NOVA National School of Public Health NOVA University Lisbon Lisbon Portugal

**Keywords:** target trial emulation, real-world evidence, risk of bias, regulatory decision-making, health technology assessment, scoping review, stakeholder survey

## Abstract

**Background:**

Target trial emulation (TTE) studies are increasingly important for regulatory decision‑making and health technology assessments (HTAs), but current risk-of-bias (RoB) tools are not specifically tailored to assess biases central to TTE studies—such as immortal time bias, misaligned eligibility criteria, and prevalent user bias. RoB assessment for TTE studies thus remains difficult, and it is unclear which tools and adaptations researchers currently use or how well they work.

**Objective:**

This study aims to analyze the RoB assessment of TTE studies at regulatory and policy decision points along the medical product life cycle by providing a systematic overview of tools developed, used, adapted, or evaluated for TTE studies and the perceived importance and feasibility of assessing TTE studies by HTA assessors, regulators, systematic reviewers, and methods experts.

**Methods:**

This is a protocol for a scoping review and online survey among systematic reviewers, methods experts, European HTA organizations, and regulators. We will collect data on the name, description, and feasibility of RoB tools used, adapted, and developed for TTE studies, as well as RoB assessment results and the RoB sources relevant for TTE studies. Two authors will use qualitative content analysis to analyze the data independently. The systematic search for published and gray literature in 4 electronic databases (PubMed, Scopus, Web of Science, and the Cochrane Library), on relevant websites (eg, Cochrane, HTA and regulatory agencies), and through reference list checking was conducted in May to June 2026.

**Results:**

As of August 25, 2026, screening is ongoing, and 78 references have been preliminarily included in the scoping review. We will analyze the scoping review and survey data and provide an overview of the types, feasibility, and assessment results of RoB tools currently used, adapted, or developed for TTE studies, as well as the RoB sources considered relevant for TTE studies by HTA assessors, regulators, systematic reviewers, and methods experts. Analysis results of the literature review and survey are expected in September 2026 and November 2026, respectively.

**Conclusions:**

The analysis of the scoping review and survey data will inform users of TTE evidence about the feasibility of RoB tools; future development and training needs; and the perceived value of assessing the RoB in HTAs, regulatory decisions, and systematic reviews.

**International Registered Report Identifier (IRRID):**

DERR1-10.2196/107499

## Introduction

Regulatory and policy decisions for pharmaceutical products rely on the assessment and integration of the best available evidence across the entire life cycle of a medicine, from research and development to marketing authorization and postauthorization monitoring. In the European Union, this involves international bodies such as the European Medicines Agency (EMA) and the European Commission, as well as national regulatory authorities and health technology assessment (HTA) organizations. Regulators such as the EMA evaluate and monitor the benefit-risk profile of medicines and issue decisions, including clinical trial authorizations, marketing authorizations, variation approvals, and suspensions. HTA bodies, on the other hand, assess comparative effectiveness, cost-effectiveness, and broader system impacts to support reimbursement, pricing, and access decisions. In addition, regulators and HTA bodies may consult pharmaceutical companies on evidentiary requirements or during the research and development phase of a new drug [1].

During marketing authorization, regulatory assessments rely mainly on evidence from preapproval clinical studies, particularly randomized controlled trials (RCTs). After authorization, regulators and HTA bodies make increasing use of postauthorization studies, routine pharmacovigilance, and other real-world data (RWD) sources to support postmarket evaluations. Evidence generated from RWD can also influence earlier decision points by prompting regulators or HTA bodies to revisit assumptions made during drug development or revise decisions on drug use, safety, or future evidence requirements [2,3]. This iterative process helps keep regulatory and policy decisions aligned with the evolving evidence base. Regulators and HTA organizations increasingly rely on real-world evidence (RWE), and methods capable of producing robust estimates from RWD have become increasingly relevant [3-7]. Within this context, observational studies that emulate a hypothetical target trial have gained prominence.

Target trial emulation (TTE) studies apply causal inference principles to RWD and can provide complementary evidence, address evidence gaps when RCTs are not feasible or available, and inform decision-makers throughout the life cycle of a medicine [8,9]. To achieve this, decision-makers must be able to appraise the credibility of the evidence from TTE studies, which requires transparent reporting and appropriate risk-of-bias (RoB) assessment. However, substantial reporting gaps were identified by a systematic review of 200 TTE studies: only 10% were prospectively registered; 37% reported relevant unmeasured confounders; 63% reported data sources; and although 96% reported treatment types, only 19%, 13%, and 12% reported dosage, duration, and frequency of the treatments, respectively [9]. These gaps reflect both limitations in the underlying data and shortcomings in reporting practices. To this end, new guidelines and recommendations specifically for TTE study conduct and reporting have been developed in recent years [10-16]. Reporting and conduct of TTE studies may thus improve over time. However, assessing the RoB may remain difficult as tools specifically for TTE studies are missing. While almost 100 RoB tools for nonrandomized studies with comparative groups exist, it is unclear whether any are detailed enough and fully suitable for evaluating TTE studies [17,18]. For example, the Cochrane RoB tool ROBINS-I (Risk of Bias in Nonrandomized Studies of Interventions) was developed for all nonrandomized studies of interventions, but immortal time bias, an important RoB in studies emulating a target trial, is not part of the assessment tool [19,20]. Currently, a new version of the ROBINS-I tool is in development to address this issue [21]. However, other adaptations may be required, such as specifically assessing the risk of selection bias and prevalent user bias arising from misaligning eligibility criteria, treatment assignment, and start of follow-up [4].

This raises a central question: which RoB tools are currently used for TTE studies, and what challenges and potential solutions arise when applying these tools in practice? Answering this will help clarify how to assess the RoB in TTE studies at regulatory and policy decision points and enable better integration of RCT and TTE evidence to inform decisions related to the safety, efficacy, cost-effectiveness, and reimbursement of medical products.

The aim of the study is to analyze the RoB assessment of TTE studies at regulatory and policy decision points along the medical product life cycle. The study will provide a systematic overview of the RoB tools developed, used, adapted, or evaluated for TTE studies in relation to 4 target groups, namely, HTA assessors, regulators, systematic reviewers, and methods experts, and assess these groups’ perceptions of the importance and feasibility of RoB assessment in TTE studies.

## Methods

### Overview

To reach our aim, we will conduct both a scoping review and an online survey to collect and analyze the relevant data. The scoping review will be conducted in accordance with the Joanna Briggs Institute methodology for scoping reviews, and reporting will follow the PRISMA-ScR (Preferred Reporting Items for Systematic Reviews and Meta-Analyses extension for Scoping Reviews) reporting guidelines [22]. At the time of protocol submission, no study activities had been initiated beyond the systematic search and study selection for the scoping review.

Throughout the project, we use and predefine the following terms: “RoB,” “RoB tool,” and “TTE.” We use the term “RoB” to refer to a systematic error that affects the internal validity of a study and can distort the results (eg, the estimated effect of an intervention). The RoB may be caused by a flaw in the study design or methods. “RoB tool” refers to both formal and informal assessments of the RoB in TTE studies. We define formal RoB tools as published and standardized instruments designed to assess the internal (and external) validity of a study using a predefined structure (eg, ROBINS-I). We will use the term “informal RoB tool” to describe any nonstandardized, internally developed, or ad hoc process used to judge the internal (and external) validity of a study without relying on a published instrument. We use the term “TTE” as described by Hernán and Robins [10].

### Data Collection

#### Scoping Review: Search Strategy

We enlisted the help of an information specialist from the University of Eastern Finland to develop and execute the search strategy across multiple databases. We completed the systematic search in May 2026 and June 2026 before submission of the protocol manuscript.

The systematic search included a search for published and gray literature. Our search strategy used English-language search terms for “risk of bias assessment” and “target trial emulation” and, thus, relied on indexed records in English. We did not specifically search for literature written in a language other than English. The TTE framework was first proposed in 2016 [23] and has grown exponentially in health science since then [8,9]. However, we did not restrict our search to the period from 2016 to the present, as we used specific search terms for TTE (Multimedia Appendices 1-4).

The search was conducted in PubMed, Scopus, Web of Science, and the Cochrane Library. In addition, we searched for gray literature in the international HTA database for HTA reports and the review by Bachinger et al [7] for included European public assessment reports. We also conducted a pragmatic search on the publicly available web pages of systematic review groups (eg, Cochrane and Campbell), European HTA and regulatory agencies, and relevant networks for methodological guidance documents that specifically address RoB assessment in TTE studies (Multimedia Appendix 5).

#### Scoping Review: Eligibility Criteria

We prespecified the inclusion of the following types of publications without language restrictions or restrictions regarding the local context of the literature (Table 1). Any systematic review that included methods to assess the RoB in TTE studies (ie, used the ROBINS-I tool for TTE studies or provided an overview of RoB tool adaptations for TTE studies) was eligible for inclusion (ie, scoping reviews, rapid reviews, and systematic reviews with or without meta-analysis). Primary studies were eligible for inclusion if they were methods papers that developed or evaluated RoB tools specifically for TTE studies (ie, development of new tools or tool adaptations and evaluation of tool usability). Furthermore, the following types of gray literature were eligible for inclusion in the scoping review: guidelines, reports, white papers, and similar publications from, for example, systematic review methods groups or HTA and regulatory bodies on RoB assessment in TTE studies.

**Table 1:** Scoping review: eligibility criteria for inclusion of literature applied during reference screening (July 2026-August 2026).

Domain	Inclusion criteria	Exclusion criteria
Study design and publication type	Protocols and full reports of any of the following types: systematic reviews (any type, ie, rapid reviews and scoping reviews with methods for RoB^a^ assessment) and methods papers or primary studies with methods for RoB assessment Gray literature: methodological guidelines, white papers, and reports published by regulatory and HTA^b^ bodies (ie, HTA reports, European public assessment reports, and general methods reports from regulatory bodies)	Opinion pieces by one author and studies without methods for RoB assessment (eg, clinical trials)
Content	Includes the description, evaluation, use, adaptation, or development of a RoB tool (eg, ROBINS-I^c^, APPRAISE^d^, or ad hoc checklists) for any TTE^e^ study (irrespective of the intervention, population, setting, and comparison)	No RoB tool (eg, assessment focused on reporting quality only, discussion of potential RoB sources, or how to benchmark against existing trial results without a method for assessing the RoB in TTE studies) RoB tool not relevant to TTE (eg, tool specifically designed for other study designs [eg, ITS^f^ or case-control])
Outcomes	Any of the following outcomes: RoB sources in TTE studies (internal validity), RoB assessment results for TTE studies (eg, high, moderate, or low), name and/or description of the RoB tool for TTE studies (eg, ROBINS-I or internal checklist), development results (eg, new tool or adaptations, such as adding a RoB domain for assessing TTE studies), or evaluation results (eg, challenges in assessing the RoB in TTE studies)	None of the listed outcomes (eg, TTE studies were included in a systematic review but the RoB was only assessed for other study designs)

^a^RoB: risk of bias.

^b^HTA: health technology assessment.

^c^ROBINS-I: Risk of Bias in Nonrandomized Studies of Interventions.

^d^APPRAISE: Appraisal of Potential for Bias in Real-World Evidence Studies.

^e^TTE: target trial emulation.

^f^ITS: interrupted time series.

#### Scoping Review: Selection of Sources of Evidence (Screening)

Screening of the search results had been initiated but not completed at the time of manuscript submission. No changes to the methods of the protocol have been made after submission. As per the protocol, screening of the search results from the electronic databases (PubMed, Scopus, Web of Science, and the Cochrane Library) was conducted in duplicate for eligibility using Covidence (Veritas Health Innovation) [24]. For gray literature, one reviewer (AK) searched and screened for eligibility, with a second reviewer (SP, CT, or MR) double-checking the results.

#### Scoping Review: Data Extraction

Data will be extracted from the full texts of the included literature in duplicate using Covidence [24] and exported to Microsoft Excel.

#### Scoping Review and Survey: Types of Data to Be Collected

##### Overview

We will collect data on the tools developed, used, adapted, or evaluated for TTE studies and the perceived importance and feasibility of assessing TTE studies by HTA assessors, regulators, systematic reviewers, and methods experts. We will not evaluate ourselves whether available RoB tools or adaptations are feasible for TTE studies (eg, available RoB assessment tools for RWE studies [25]). We will collect the following data from the literature included in the scoping review and from survey participants.

##### Background Information

From the included literature, we will collect the following metadata: first author, title, aim, year of publication, reference, and publication type (systematic review, methods paper, or gray literature). From survey participants, we will collect the following background information: stakeholder group and experience with RoB and TTE.

##### Available RoB Tools for TTE Studies

We will collect data about RoB tool names, mode (published or developed, in development, or adapted), RoB tool domains, study designs the tool is intended for, year of tool development, source, and authors and/or developers of the tool.

##### Adaptations to Existing Tools

We will collect the names of, reasons for, and description of adaptations to available RoB tools (solutions).

##### RoB Sources in TTE Studies

We will collect data on the name and description of the RoB sources.

##### Feasibility of RoB Tools for TTE Studies

We will include any outcome measurement, including opinions, experiences, and measurement results. We will include all areas of feasibility as defined by Bowen et al [26], including acceptability (ie, individuals’ reactions to the RoB tool for TTE studies), practicality (ie, the extent to which the RoB in TTE studies can be assessed and challenges such as time restrictions), and demand (ie, opinions on the importance of assessing the RoB in TTE studies to support drug‑related, evidence‑informed regulatory and policy decision-making—both in general and with respect to the depth of assessment needed).

##### Reliability of RoB Tools for TTE Studies

We will include any outcome measurement, including opinions, experiences, and measurement results, such as interrater reliability.

#### Survey: Development of the Questionnaire

Two researchers (AK and CT) will develop a first draft of the survey questionnaire based on the aims and outcomes specified in the protocol. The draft will be shared with the coauthors of this project to possibly test the survey and provide comments. Possible response formats will include open-ended questions, matrices, and other formats.

The survey will be pilot-tested with a minimum of 1 and a maximum of 3 persons from each stakeholder group depending on availability and successful recruitment. The survey will be adapted accordingly to address the feedback of the pilot test (eg, regarding clarity, content, layout, and logic of the survey). The final version will be approved by means of consensus among the author group.

#### Survey: Eligibility Criteria and Recruitment

In line with the focus of our study, the target population will include people who either make or support regulatory and policy decisions, such as authorization, reimbursement, and coverage decisions.

For the survey, we will thus actively recruit stakeholders belonging to one or more of the following target groups: HTA assessors, regulators, systematic reviewers, and methods experts. HTA assessors are defined as members of and contributors to HTA bodies (eg, health economists, epidemiologists or biostatisticians, clinicians or pharmacists, systematic review and evidence synthesis specialists, and public health or policy analysts). Regulators are defined as members of and contributors to regulatory bodies (eg, clinical assessors [physicians, pharmacists, and biomedical scientists], statisticians, epidemiologists, pharmacologists, toxicologists, health economists, regulatory scientists, and policy specialists). Systematic reviewers are defined as authors of systematic reviews (eg, Cochrane systematic review authors). Methods experts are defined as authors of or members of working groups responsible for developing RoB tools for nonrandomized studies with comparative groups.

We will neither actively recruit nor actively exclude stakeholders that do not belong to our target groups, such as persons working in the pharmacological industry, journal editors, and patient representatives. Survey participants will be asked to self-identify and specify the stakeholder group they identify with (eg, “HTA assessor,” “regulator,” “systematic reviewer,” “methods expert,” and “other please specify”).

Participants will be recruited as follows.

HTA assessors and regulators will be invited through the Portuguese National Authority of Medicines and Health Products (Infarmed) and the German Federal Institute for Drugs and Medical Devices (BfArM) via relevant HTA and regulatory networks.

Systematic reviewers will be invited directly via email. As part of the protocol, we compiled a list of email contact information of potential survey participants by conducting a literature search for any systematic reviews that included TTE studies. No language or time restrictions were applied to the search strategy. Our search strategy used English-language search terms for “target trial emulation” and the publication type “systematic review” and, thus, relied on English‑indexed records. All search results were uploaded to separate Zotero (Corporation for Digital Scholarship) and Covidence files. We used a Java script within Zotero and the automated structured template in Covidence with automatic extraction suggestions (AI feature) to collect the following information in Microsoft Excel: author names and affiliations, email contact information, publication title, year, journal, and digital object identifier. Each automatic extraction suggestion in Covidence was verified by one reviewer as the setup requires reviewers to accept or reject each suggestion individually.

Methods experts will be identified and invited as methods experts if they are listed in the systematic overview by Siedler et al [18] or are involved in the development of additional eligible tools identified through the systematic search conducted as part of the scoping review. In addition, the invitation to the survey will be circulated via relevant methods working groups (ie, from Cochrane, Campbell, and RWE or RWD collaboration networks).

The survey will be conducted in September 2026 and October 2026 using the EUSurvey platform [27], including sending direct invitations and reminders to participants.

### Data Analysis

Two researchers will independently analyze the collected data from the scoping review and survey. We will use qualitative content analysis methods to systematically reduce the data to codes and categories using the framework method [28]. The framework method enables the use of codes that were defined before data collection, as well as codes derived from the data. We will develop a set of predefined codes and new codes when appropriate (Table 2). We will analyze the survey data separately for different stakeholder groups to compare results across stakeholder groups (eg, HTA assessors, regulators, systematic reviewers, and methods experts).

**Table 2:** Scoping review and survey: preliminary list of predefined codes for data analysis (planned for September 2026-November 2026).

Codes	Description
Sources of bias	Sources of bias in TTE^a^ study results identified as critical, key concerns, or main sources of bias by HTA^b^ assessors, regulators, systematic reviewers, and methods experts, including RoB^c^ domains
Availability of RoB tools	Tools (planned to be) developed, used, and/or adopted for or by HTA assessors, regulators, systematic reviewers, and methods experts for assessing the RoB in TTE studies
RoB tool adaptation	(Planned) adaptations to available RoB tools by HTA assessors, regulators, systematic reviewers, and methods experts for assessing TTE study results
Feasibility of RoB tools	Opinions or measurements regarding the feasibility of available tools for assessing the RoB in TTE study results by HTA assessors, regulators, systematic reviewers, and methods experts (ie, the extent to which the RoB can be assessed, challenges such as time restrictions, and the acceptance of tools)
Reliability of RoB tools	Validity of the reviewers’ results (ie, interrater reliability)
Importance of RoB assessment in TTE studies for EiD^d^	Opinions regarding the importance of accurate RoB assessment of TTE studies for making an EiD at critical decision points along the life cycle of a medical product, including marketing authorization scientific opinion, marketing authorization commission decision, HTA, and reimbursement decisions

^a^TTE: target trial emulation.

^b^HTA: health technology assessment.

^c^RoB: risk of bias.

^d^EiD: evidence-informed decision.

### Ethical Considerations

According to the Finnish National Board on Research Integrity TENK guidelines of 2019, an ethical review statement from a human sciences ethics committee is not required for this study [29]. The scoping review will use publicly available literature and does not involve human participants directly. The online survey will focus on professional practices and experiences and will follow the principle of informed consent. Participation is entirely voluntary, and no incentives will be offered. Before accessing the questionnaire, participants will be provided with an online information sheet outlining the study purpose, procedures, expected time commitment, potential risks and benefits, data handling practices, and contact details for the research team. Informed consent will be obtained electronically. Participants may withdraw at any point before submitting the survey by closing their browser window. Because no identifiable information is collected, withdrawal after submission will not be possible.

## Results

The systematic search was conducted in May 2026 and June 2026 (Multimedia Appendices 1-5). Screening of the search results had been initiated but not completed at the time of manuscript submission (July 20, 2026). As of July 20, 2026, screening of the primary search results from electronic databases resulted in 62 included articles (Figure 1). We contacted Bachinger et al [7] to retrieve the list of 39 EMA reports containing RWE, none of which fulfilled our eligibility criteria. Additionally, the search on the websites of 60 HTA and regulatory agencies and 9 relevant networks retrieved 278 gray literature references.

As of August 25, 2026, 255 of 278 gray literature references had been screened in duplicate, 16 of which will be included for data extraction. The preliminary total of included references was 78 as of August 25, 2026. The survey will be conducted in September 2026 and October 2026. Analysis results of the literature review and survey are expected in September 2026 and November 2026, respectively.

**Figure 1:**
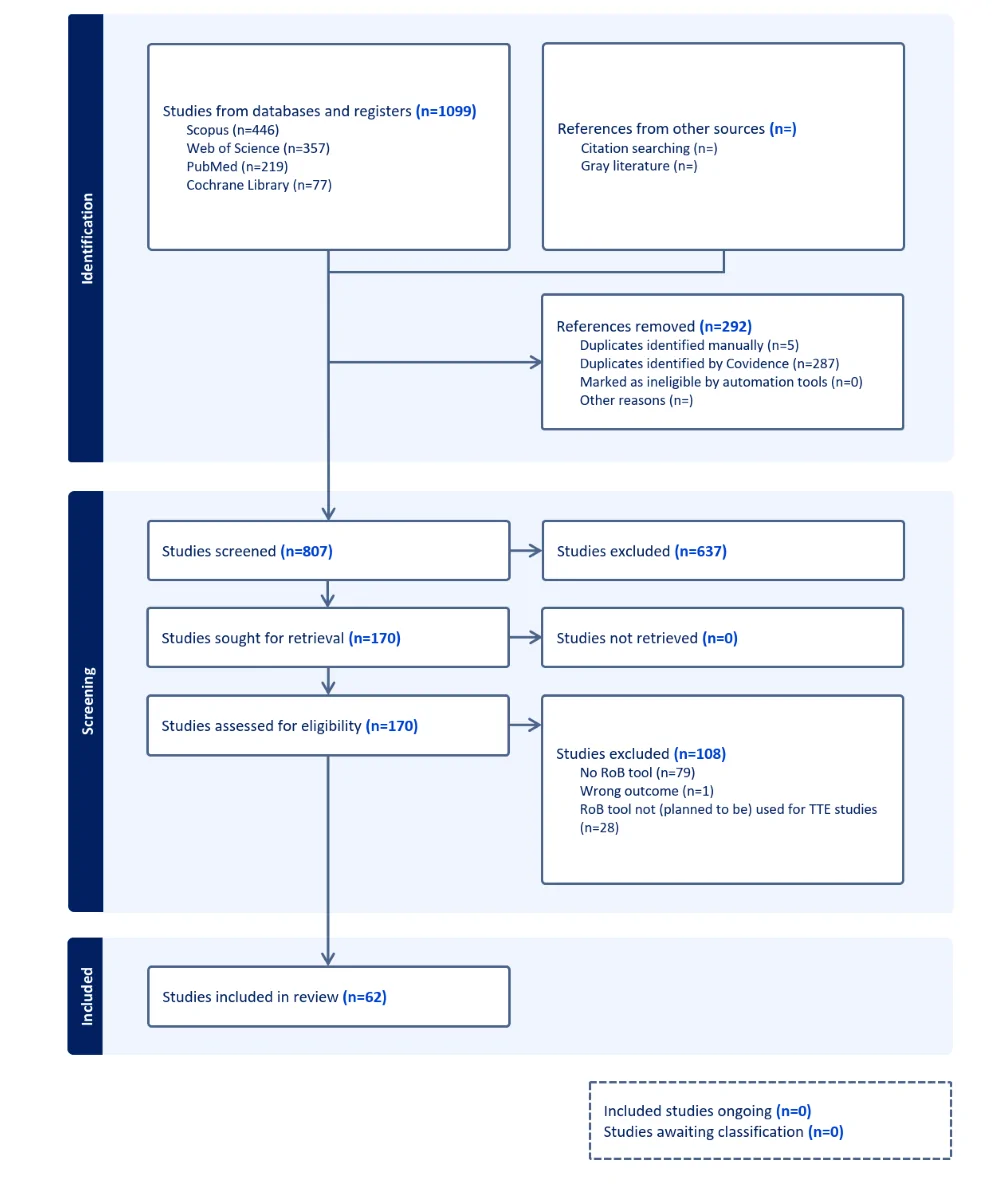
Scoping review: PRISMA (Preferred Reporting Items for Systematic Reviews and Meta-Analyses) flowchart—search and screening results of electronic databases (conducted in May 2026-July 2026). RoB: risk of bias; TTE: target trial emulation.

## Discussion

### Anticipated Findings

Principal results are expected to list the RoB tools specifically developed, used, adapted, or evaluated for TTE studies (eg, ROBINS-I and ROBINS-E [Risk of Bias in Nonrandomized Studies of Exposure] tool) and the perceived importance and feasibility of assessing the RoB in TTE studies by HTA assessors, regulators, systematic reviewers, and methods experts as defined by Bowen et al [26] (eg, practicality of the tool). We expect that the findings will provide new insights into which tools are available to different stakeholder groups. In addition, the findings will inform future development needs (eg, whether new tools or better guidance documents for reviewers are needed to increase practicality). The results will also identify the training needs of users of RWE with experience in HTA, medicine regulation, or systematic review conduct and the dissemination needs related to new or existing RoB tools and guidance among these user groups. The findings may show a difference between systematic reviewers, HTA assessors, and regulators in how RoB is assessed and how different tools are valued and needed to perform their respective tasks. The findings may clarify whether the use of different tools could be harmonized across different stakeholder groups. A common tool and process for assessing the validity of TTE evidence will help streamline decision-making and communication between different stakeholders.

Our findings are expected to complement and extend previous research on the evidence from TTE studies, which has primarily focused on methodological applications and reporting practices rather than on the assessment of the RoB. Previous systematic reviews have highlighted inconsistent reporting of TTE studies and limited transparency in addressing biases such as selection and immortal time bias while providing little detail on how RoB is assessed [9,30]. At the same time, methodological frameworks (eg, the original TTE framework, HARPER [Harmonized Protocol Template to Enhance Reproducibility], and PRINCIPLED [Process Guide for Inferential Studies Using Healthcare Data From Routine Clinical Practice to Evaluate Causal Effects of Drugs]) and reporting guidelines (eg, STaRT-RWE [Structured Template for Planning and Reporting on the Implementation of Real-World Evidence Studies], RECORD-PE [Reporting of Studies Conducted Using Observational Routinely Collected Health Data statement for pharmacoepidemiology], TARGET [Transparent Reporting of Observational Studies Emulating a Target Trial], and TITAN [Transparency in the Reporting of Artificial Intelligence]) offer structured guidance for study design and transparency but do not address how RoB should be evaluated in practice [9,10,12-16,31]. Others have focused on the context of HTA and highlighted challenges related to the integration of RWD [3-5]. Overall, while these studies provide valuable insights into where and how TTE has been applied, they do not systematically examine how RoB in TTE studies is assessed, nor do they address methodological approaches for integrating evidence from RCTs and TTE studies. In this context, our study will address an important gap by focusing specifically on RoB assessment practices for TTE studies and their use in applied evidence synthesis contexts such as HTA.

### Strengths and Limitations

We anticipate that a key limitation of our study design will be the use of English-language search terms, which may lead to incomplete retrieval of relevant studies and gray literature published in other languages. However, this limitation is mitigated by searching multiple sources for published and gray literature (including the international HTA database, multiple electronic scientific databases, relevant websites of national and international organizations, reference screening, and survey data), thereby reducing reliance on any single retrieval strategy. In addition, many studies indexed in electronic databases provide English-language abstracts and keywords, and the term “target trial emulation” is often used in English regardless of the publication language. Consequently, while the overall set of identified studies may not be fully comprehensive, it is unlikely that the omission of individual studies or gray literature reports would substantially alter the conclusions.

### Conclusions

This paper presents the protocol for a scoping review and survey aimed at analyzing the RoB assessment of TTE studies at regulatory and policy decision points along the medical product life cycle. In the conclusions of this study, we will use our findings to help clarify when, how, and why the RoB in TTE studies is or could be assessed to inform decisions related to the safety, efficacy, cost-effectiveness, and reimbursement of medical products. We will further use our findings to inform future studies. Possible future research directions may be follow-up studies to deepen our understanding regarding, for example, the value of RoB tools at regulatory and policy decision points or new studies that focus on other stakeholder groups and time points, for example, critical decision points for industry during research and development.

## Appendix

Multimedia Appendix 1 Scoping review search strategy—PubMed (May 13, 2026; 219 results).

Multimedia Appendix 2 Scoping review search strategy—Scopus (May 13, 2026; 446 results).

Multimedia Appendix 3 Scoping review search strategy—Web of Science (May 13, 2026; 357 results).

Multimedia Appendix 4 Scoping review search strategy—Cochrane Library (May 13, 2026; 77 results).

Multimedia Appendix 5 Scoping review search strategy—relevant websites of health technology assessment bodies, regulatory agencies, and networks.

## Abbreviations

EMA: European Medicines Agency
HARPER: Harmonized Protocol Template to Enhance Reproducibility
HTA: health technology assessment
PRINCIPLED: Process Guide for Inferential Studies Using Healthcare Data From Routine Clinical Practice to Evaluate Causal Effects of Drugs
PRISMA-ScR: Preferred Reporting Items for Systematic Reviews and Meta-Analyses extension for Scoping Reviews
RCT: randomized controlled trial
RECORD-PE: Reporting of Studies Conducted Using Observational Routinely Collected Health Data statement for pharmacoepidemiology
RoB: risk of bias
ROBINS-E: Risk of Bias in Nonrandomized Studies of Exposure
ROBINS-I: Risk of Bias in Nonrandomized Studies of Interventions
RWD: real-world data
RWE: real-world evidence
STaRT-RWE: Structured Template for Planning and Reporting on the Implementation of Real-World Evidence Studies
TARGET: Transparent Reporting of Observational Studies Emulating a Target Trial
TITAN: Transparency in the Reporting of Artificial Intelligence
TTE: target trial emulation

## Data Availability

The authors will ensure that the research data are accessible (eg, uploaded as multimedia appendix files). Datasets will be either deposited or made available to readers in one or more of the following ways: data will be deposited in publicly available repositories (where available and appropriate); data will be supplied in supporting files available for download along with the published manuscript; data will be presented in the main manuscript; or data will be made available on request from the authors.
