# Supplementary material for: Risk-of-Bias Assessment of Target Trial Emulation Studies at Regulatory and Policy Decision Points Along the Medical Product Life Cycle: Protocol for a Scoping Review and Stakeholder Survey
Source: JMIR Res Protoc. 2026 Oct 27;15:e107499. doi: 10.2196/107499 (PMC13752585; doi:10.2196/107499)
Supplement: Supplementary file 1 [file resprot_v15i1e107499_app1.docx]

Table S1 Scoping review - search strategy_Pubmed 13.05.2026 (219 results)

| #5 | (#1 OR #2 OR #3) AND #4 | [219](https://pubmed.ncbi.nlm.nih.gov/?term=longquery33c6f042c9c77dfd27fb&sort=pubdate&ac=no) |
| --- | --- | --- |
| #4 | "target trial*"[All Fields] OR "emulat* trial*"[All Fields] OR "trial emulation*"[All Fields] OR "hypothetical trial*"[All Fields] OR "trial replicat*"[All Fields] | [2,306](https://pubmed.ncbi.nlm.nih.gov/?term=%28%22target+trial%2A%22%5BAll+Fields%5D+OR+%22emulat%2A+trial%2A%22%5BAll+Fields%5D+OR+%22trial+emulation%22%5BAll+Fields%5D+OR+%22hypothetical+trial%2A%22%5BAll+Fields%5D+OR+%22trial+replicat%2A%22%5BAll+Fields%5D%29&sort=pubdate&ac=no) |
| #3 | "APPRAISE"[All Fields] OR "AXIS tool"[All Fields] OR "BEES-C"[All Fields] OR "BEME"[All Fields] OR "CCAT"[All Fields] OR "CEBMH"[All Fields] OR "CLEAR NPT"[All Fields] OR "Coleman Methodology Score"[All Fields] OR "CSCEDARS"[All Fields] OR "CTAM"[All Fields] OR "Downs and Black"[All Fields] OR "Downs&Black"[All Fields] OR "Downs&Black"[All Fields] OR "Epidemiological Appraisal Instrument"[All Fields] OR "Evidence Project Tool"[All Fields] OR "GRACE"[All Fields] OR "Heyland Methodological Quality Score"[All Fields] OR "Jadad"[All Fields] OR "Jadad Scale"[All Fields] OR "JBI"[All Fields] OR "Maastricht-Amsterdam list"[All Fields] OR "MINCIR"[All Fields] OR "MINORS"[All Fields] OR "MMAT"[All Fields] OR "MQRS"[All Fields] OR "nih quality assessment tool*"[All Fields] OR "NICMAN"[All Fields] OR "NICE tool"[All Fields] OR "Newcastle-Ottawa Scale"[All Fields] OR "NOS scale"[All Fields] OR "OHAT"[All Fields] OR "ORBIT"[All Fields] OR "OSQE"[All Fields] OR "Oxford Pain Validity"[All Fields] OR "PEDro"[All Fields] OR "Q-Coh"[All Fields] OR "Q-Genie"[All Fields] OR "QLADS"[All Fields] OR "QualSyst tool"[All Fields] OR "RCT-PQRS"[All Fields] OR "ROB 2"[All Fields] OR "RoB 2.0"[All Fields] OR "ROBINS-I"[All Fields] OR "ROBINS-E"[All Fields] OR "RoBANS"[All Fields] OR "SIGN"[All Fields] OR "SURE"[All Fields] OR "TESTEX"[All Fields] | [304,441](https://pubmed.ncbi.nlm.nih.gov/?term=%22APPRAISE%22%5BAll+Fields%5D+OR+%22AXIS+tool%22%5BAll+Fields%5D+OR+%22BEES-C%22%5BAll+Fields%5D+OR+%22BEME%22%5BAll+Fields%5D+OR+%22CCAT%22%5BAll+Fields%5D+OR+%22CEBMH%22%5BAll+Fields%5D+OR+%22CLEAR+NPT%22%5BAll+Fields%5D+OR+%22Coleman+Methodology+Score%22%5BAll+Fields%5D+OR+%22CSCEDARS%22%5BAll+Fields%5D+OR+%22CTAM%22%5BAll+Fields%5D+OR+%22Downs+and+Black%22%5BAll+Fields%5D+OR+%22Downs+%26+Black%22%5BAll+Fields%5D+OR+%22Downs%26Black%22%5BAll+Fields%5D+OR+%22Epidemiological+Appraisal+Instrument%22%5BAll+Fields%5D+OR+%22Evidence+Project+Tool%22%5BAll+Fields%5D+OR+%22GRACE%22%5BAll+Fields%5D+OR+%22Heyland+Methodological+Quality+Score%22%5BAll+Fields%5D+OR+%22Jadad%22%5BAll+Fields%5D+OR+%22Jadad+Scale%22%5BAll+Fields%5D+OR+%22JBI%22%5BAll+Fields%5D+OR+%22Maastricht-Amsterdam+list%22%5BAll+Fields%5D+OR+%22MINCIR%22%5BAll+Fields%5D+OR+%22MINORS%22%5BAll+Fields%5D+OR+%22MMAT%22%5BAll+Fields%5D+OR+%22MQRS%22%5BAll+Fields%5D+OR+%22nih+quality+assessment+tool%2A%22%5BAll+Fields%5D+OR+%22NICMAN%22%5BAll+Fields%5D+OR+%22NICE+tool%22%5BAll+Fields%5D+OR+%22Newcastle-Ottawa+Scale%22%5BAll+Fields%5D+OR+%22NOS+scale%22%5BAll+Fields%5D+OR+%22OHAT%22%5BAll+Fields%5D+OR+%22ORBIT%22%5BAll+Fields%5D+OR+%22OSQE%22%5BAll+Fields%5D+OR+%22Oxford+Pain+Validity%22%5BAll+Fields%5D+OR+%22PEDro%22%5BAll+Fields%5D+OR+%22Q-Coh%22%5BAll+Fields%5D+OR+%22Q-Genie%22%5BAll+Fields%5D+OR+%22QLADS%22%5BAll+Fields%5D+OR+%22QualSyst+tool%22%5BAll+Fields%5D+OR+%22RCT-PQRS%22%5BAll+Fields%5D+OR+%22ROB+2%22%5BAll+Fields%5D+OR+%22RoB+2.0%22%5BAll+Fields%5D+OR+%22ROBINS-I%22%5BAll+Fields%5D+OR+%22ROBINS-E%22%5BAll+Fields%5D+OR+%22RoBANS%22%5BAll+Fields%5D+OR+%22SIGN%22%5BAll+Fields%5D+OR+%22SURE%22%5BAll+Fields%5D+OR+%22TESTEX%22%5BAll+Fields%5D&sort=pubdate&ac=no) |
| #2 | "bias"[MeSH Terms] OR "Selection Bias"[MeSH Terms] OR "risk of bias"[All Fields] OR "bias analys*"[All Fields] OR "bias apprais*"[All Fields] OR "bias assess*"[All Fields] OR "bias checklist*"[All Fields] OR "bias evaluat*"[All Fields] OR "bias examinat*"[All Fields] OR "bias instrument*"[All Fields] OR "bias rate*"[All Fields] OR "bias rating*"[All Fields] OR "bias scoring*"[All Fields] OR "bias score*"[All Fields] OR "bias test*"[All Fields] OR "bias tool*"[All Fields] OR "quality analys*"[All Fields] OR "quality apprais*"[All Fields] OR "quality assess*"[All Fields] OR "quality checklist*"[All Fields] OR "quality evaluat*"[All Fields] OR "quality examinat*"[All Fields] OR "quality instrument*"[All Fields] OR "quality rate*"[All Fields] OR "quality rating*"[All Fields] OR "quality scoring*"[All Fields] OR "quality score*"[All Fields] OR "quality tool*"[All Fields] OR "quality test*"[All Fields] OR "critical* appraisal*"[All Fields] OR "critical* assess*"[All Fields] OR "methodolog* apprais*"[All Fields] OR "methodolog* assess*"[All Fields] OR "method* checklist*"[All Fields] OR "methodological rigour"[All Fields] OR "method* quality"[All Fields] OR "credibility assess*"[All Fields] OR "credibility apprais*"[All Fields] OR "credibility checklist*"[All Fields] OR "study apprais*"[All Fields] OR "validity assess*"[All Fields] OR "validity apprais*"[All Fields] OR "validity* checklist*"[All Fields] | [270,334](https://pubmed.ncbi.nlm.nih.gov/?term=%22bias%22%5BMeSH+Terms%5D+OR+%22Selection+Bias%22%5BMeSH+Terms%5D+%0AOR+%22risk+of+bias%22%5BAll+Fields%5D+%0AOR+%0A%22bias+analys%2A%22%5BAll+Fields%5D+OR+%22bias+apprais%2A%22%5BAll+Fields%5D+OR+%E2%80%9Cbias+assess%2A%22%5BAll+Fields%5D+OR+%22bias+checklist%2A%22%5BAll+Fields%5D+OR+%22bias+evaluat%2A%22%5BAll+Fields%5D+OR+%22bias+examinat%2A%22%5BAll+Fields%5D+OR+%22bias+instrument%2A%22%5BAll+Fields%5D+OR+%22bias+rate%2A%22%5BAll+Fields%5D+OR+%22bias+rating%2A%22%5BAll+Fields%5D+OR+%22bias+scoring%2A%22%5BAll+Fields%5D+OR+%22bias+score%2A%22%5BAll+Fields%5D+OR+%22bias+test%2A%22%5BAll+Fields%5D+OR+%22bias+tool%2A%22%5BAll+Fields%5D%0AOR+%0A%22quality+analys%2A%22%5BAll+Fields%5D+OR+%22quality+apprais%2A%22%5BAll+Fields%5D+OR+%22quality+assess%2A%22%5BAll+Fields%5D+OR+%22quality+checklist%2A%22%5BAll+Fields%5D+OR+%22quality+evaluat%2A%22%5BAll+Fields%5D+OR+%22quality+examinat%2A%22%5BAll+Fields%5D+OR+%22quality+instrument%2A%22%5BAll+Fields%5D+OR+%22quality+rate%2A%22%5BAll+Fields%5D+OR+%22quality+rating%2A%22%5BAll+Fields%5D+OR+%22quality+scoring%2A%22%5BAll+Fields%5D+OR+%22quality+score%2A%22%5BAll+Fields%5D+OR+%22quality+tool%2A%22%5BAll+Fields%5D+OR+%22quality+test%2A%22%5BAll+Fields%5D%0AOR+%0A%22critical%2A+appraisal%2A%22%5BAll+Fields%5D+OR+%22critical%2A+assess%2A%22%5BAll+Fields%5D+%0AOR+%0A%22methodolog%2A+apprais%2A%22%5BAll+Fields%5D+OR+%22methodolog%2A+assess%2A%22%5BAll+Fields%5D+OR+%22method%2A+checklist%2A%22%5BAll+Fields%5D+OR+%E2%80%9Cmethodological+rigour%E2%80%9D%5B+All+Fields%5D+OR+%22method%2A+quality%22%5BAll+Fields%5D%0AOR+%0A%22credibility+assess%2A%22%5BAll+Fields%5D+OR+%22credibility+apprais%2A%22%5BAll+Fields%5D+OR+%22credibility+checklist%2A%22%5BAll+Fields%5D%0AOR+%0A%22study+apprais%2A%22%5BAll+Fields%5D%0AOR+%0A%22validity+assess%2A%22%5BAll+Fields%5D+OR+%22validity+apprais%2A%22%5BAll+Fields%5D+OR+%22validity%2A+checklist%2A%22%5BAll+Fields%5D&sort=pubdate&ac=no) |
| #1 | ("Research Design"[MeSH Terms] OR "Evidence-Based Medicine"[MeSH Terms] OR "Systematic Reviews as Topic"[MeSH] OR "Meta-Analysis as Topic"[MeSH] OR "Evidence-Based Practice"[MeSH] OR "Quality Assurance, Health Care"[MeSH Terms] OR "Checklist"[MeSH] OR "Surveys and Questionnaires"[MeSH] OR "appraisal checklist*"[All Fields] OR "appraisal tool*"[All Fields] OR "assessment checklist*"[All Fields] OR "assessment tool*"[All Fields] OR "evaluation checklist*"[All Fields] OR "evaluation tool*"[All Fields])  AND  ("bias"[MeSH Terms] OR "Selection Bias"[Mesh Terms] OR "Confounding Factors, Epidemiologic"[Mesh] OR "reproducibility of results"[MeSH Terms] OR "method* quality"[All Fields] OR "method* flaw*" OR "method* error*" OR "study quality"[All Fields] OR "study limitation*"[All Fields] OR "inconsistency"[All Fields] OR "indirectness"[All Fields] OR "imprecision"[All Fields] OR "bias*"[All Fields] OR "validity"[All Fields] OR “confound*"[All Fields] OR "systematic error"[All Fields] OR "reliability"[All Fields] OR “credib*”[All Fields]) | [255,542](https://pubmed.ncbi.nlm.nih.gov/?term=%28%28%22Research+Design%22%5BMeSH+Terms%5D+OR+%22Evidence-Based+Medicine%22%5BMeSH+Terms%5D+OR+%22Systematic+Reviews+as+Topic%22%5BMeSH%5D+OR+%22Meta-Analysis+as+Topic%22%5BMeSH%5D+OR+%22Evidence-Based+Practice%22%5BMeSH%5D+OR+%22Quality+Assurance%2C+Health+Care%22%5BMeSH+Terms%5D+OR+%22Checklist%22%5BMeSH%5D+OR+%22Surveys+and+Questionnaires%22%5BMeSH%5D+OR+%22appraisal+checklist%2A%22%5BAll+Fields%5D+OR+%22appraisal+tool%2A%22%5BAll+Fields%5D+OR+%22assessment+checklist%2A%22%5BAll+Fields%5D+OR+%22assessment+tool%2A%22%5BAll+Fields%5D+OR+%22evaluation+checklist%2A%22%5BAll+Fields%5D+OR+%22evaluation+tool%2A%22%5BAll+Fields%5D%29+%0AAND%0A%28%22bias%22%5BMeSH+Terms%5D+OR+%22Selection+Bias%22%5BMesh+Terms%5D+OR+%22Confounding+Factors%2C+Epidemiologic%22%5BMesh%5D+OR+%22reproducibility+of+results%22%5BMeSH+Terms%5D+OR+%22method%2A+quality%22%5BAll+Fields%5D+OR+%22method%2A+flaw%2A%22+OR+%22method%2A+error%2A%22+OR+%22study+quality%22%5BAll+Fields%5D+OR+%22study+limitation%2A%22%5BAll+Fields%5D+OR+%22inconsistency%22%5BAll+Fields%5D+OR+%22indirectness%22%5BAll+Fields%5D+OR+%22imprecision%22%5BAll+Fields%5D+OR+%22bias%2A%22%5BAll+Fields%5D+OR+%22validity%22%5BAll+Fields%5D+OR+%E2%80%9Cconfound%2A%22%5BAll+Fields%5D+OR+%22systematic+error%22%5BAll+Fields%5D+OR+%22reliability%22%5BAll+Fields%5D+OR+%E2%80%9Ccredib%2A%E2%80%9D%5BAll+Fields%5D%29%29&sort=pubdate&ac=no) |
