# Supplementary material for: Risk-of-Bias Assessment of Target Trial Emulation Studies at Regulatory and Policy Decision Points Along the Medical Product Life Cycle: Protocol for a Scoping Review and Stakeholder Survey
Source: JMIR Res Protoc. 2026 Oct 27;15:e107499. doi: 10.2196/107499 (PMC13752585; doi:10.2196/107499)
Supplement: Supplementary file 2 [file resprot_v15i1e107499_app2.docx]

Table S2 Scoping review - search strategy_Scopus 13.05.2026 (446 results)

| #5 | (#1 OR #2 OR #3) AND #4 | 446 |
| --- | --- | --- |
| #4 | TITLE-ABS-KEY("target trial*" OR "emulat* trial*" OR "trial emulation*" OR "hypothetical trial*" OR "trial replicat*") | 2,617 |
| #3 | ALL("APPRAISE" OR "AXIS tool" OR "BEES-C" OR "BEME" OR "CCAT" OR "CEBMH" OR "CLEAR NPT" OR "Coleman Methodology Score" OR "CSCEDARS" OR "CTAM" OR "Downs and Black" OR "Downs&Black" OR "Downs&Black" OR "Epidemiological Appraisal Instrument" OR "Evidence Project Tool" OR "GRACE" OR "Heyland Methodological Quality Score" OR "Jadad" OR "Jadad Scale" OR "JBI" OR "Maastricht-Amsterdam list" OR "MINCIR" OR "MINORS" OR "MMAT" OR "MQRS" OR "NIH quality assessment tool*" OR "NICMAN" OR "NICE tool" OR "Newcastle-Ottawa Scale" OR "NOS scale" OR "OHAT" OR "ORBIT" OR "OSQE" OR "Oxford Pain Validity" OR "PEDro" OR "Q-Coh" OR "Q-Genie" OR "QLADS" OR "QualSyst tool" OR "RCT-PQRS" OR "ROB 2" OR "RoB 2.0" OR "ROBINS-I" OR "ROBINS-E" OR "RoBANS" OR "SIGN" OR "SURE" OR "TESTEX") | 5,805,719 |
| #2 | TITLE-ABS-KEY("bias analys*" OR "bias apprais*" OR "bias assess*" OR "bias checklist*" OR "bias evaluat*" OR "bias examinat*" OR "bias instrument*" OR "bias rate*" OR "bias rating*" OR "bias scoring*" OR "bias score*" OR "bias test*" OR "bias tool*" OR “risk of bias” OR "quality analys*" OR "quality apprais*" OR "quality assess*" OR "quality checklist*" OR "quality evaluat*" OR "quality examinat*" OR "quality instrument*" OR "quality rate*" OR "quality rating*" OR "quality scoring*" OR "quality score*" OR "quality tool*" OR "quality test*" OR "critical appraisal*" OR "critical assess*" OR "methodolog* apprais*" OR "methodolog* assess*" OR "method* checklist*" OR "methodological rigour" OR "method* quality" OR "credibility assess*" OR "credibility apprais*" OR "credibility checklist*" OR "study apprais*" OR "validity assess*" OR "validity apprais*" OR "validity checklist*") | 305,748 |
| #1 | TITLE-ABS-KEY( "research design" OR "study design" OR "methodological design" OR "research method*" OR "experimental design" OR "research plan*" OR "evidence based medicine" OR "evidence-based medicine" OR "EBM" OR "evidence based practice" OR "evidence-based practice" OR "EBP" OR "evidence informed decision*" OR "evidence based decision*" OR "evidence-based decision*" OR "EiD" OR "evidence informed practice" OR "systematic review*" OR "systematic overview*" OR "literature review*" OR "evidence synthesis" OR “review method*” OR "quantitative synthesis" OR "qualitative synthesis" OR "meta analysis" OR "meta-analysis" OR "meta analytic*" OR "quality assurance" OR "health care quality" OR "healthcare quality" OR "quality improvement" OR "quality indicators" OR "quality management" OR "quality measurement" OR "performance improvement" OR "quality management" OR "clinical audit*" OR "medical audit*" OR "nurs* audit*" OR "utili?ation review" OR guideline* OR checklist* OR "check list*" OR survey* OR questionnaire* OR "appraisal checklist*" OR "appraisal tool*" OR "assessment checklist*" OR "assessment tool*" OR "evaluation checklist*" OR "evaluation tool*")  AND  ALL( "method* quality" OR "method* flaw*" OR "method* error*" OR "study quality" OR "study limitation*" OR "inconsistency" OR "indirectness" OR "imprecision" OR "bias*" OR "validity" OR confound* OR "systematic error" OR "reliability" OR credib*) | 228,731 |
