# Supplementary material for: Risk-of-Bias Assessment of Target Trial Emulation Studies at Regulatory and Policy Decision Points Along the Medical Product Life Cycle: Protocol for a Scoping Review and Stakeholder Survey
Source: JMIR Res Protoc. 2026 Oct 27;15:e107499. doi: 10.2196/107499 (PMC13752585; doi:10.2196/107499)
Supplement: Supplementary file 3 [file resprot_v15i1e107499_app3.docx]

Table S3 Scoping review – search strategy_Web of Science 13.05.2026 (357 results)

| #1 | ((TS=("research design" OR "study design" OR "methodological design" OR "research method*" OR "experimental design" OR "research plan*" OR "evidence based medicine" OR "evidence based medicine" OR "EBM" OR "evidence based practice" OR "evidence based practice" OR "EBP" OR "evidence informed decision*" OR "evidence based decision*" OR "evidence based decision*" OR "EiD" OR "evidence informed practice" OR "systematic review*" OR "systematic overview*" OR "literature review*" OR "evidence synthesis" OR "review method*" OR "quantitative synthesis" OR "qualitative synthesis" OR "meta analysis" OR "meta analysis" OR "meta analytic*" OR "quality assurance" OR "health care quality" OR "healthcare quality" OR "quality improvement" OR "quality indicators" OR "quality management" OR "quality measurement" OR "performance improvement" OR "quality management" OR "clinical audit*" OR "medical audit*" OR "nurs* audit*" OR "utili?ation review" OR guideline* OR checklist* OR "check list*" OR survey* OR questionnaire* OR "appraisal checklist*" OR "appraisal tool*" OR "assessment checklist*" OR "assessment tool*" OR "evaluation checklist*" OR "evaluation tool*") AND TS=("method* quality" OR "method* flaw*" OR "method* error*" OR "study quality" OR "study limitation*" OR "inconsistency" OR "indirectness" OR "imprecision" OR "bias*" OR "validity" OR confound* OR "systematic error" OR "reliability" OR credib*)) OR TS=("bias analys*" OR "bias apprais*" OR "bias assess*" OR "bias checklist*" OR "bias evaluat*" OR "bias examinat*" OR "bias instrument*" OR "bias rate*" OR "bias rating*" OR "bias scoring*" OR "bias score*" OR "bias test*" OR "bias tool*" OR "risk of bias" OR "quality analys*" OR "quality apprais*" OR "quality assess*" OR "quality checklist*" OR "quality evaluat*" OR "quality examinat*" OR "quality instrument*" OR "quality rate*" OR "quality rating*" OR "quality scoring*" OR "quality score*" OR "quality tool*" OR "quality test*" OR "critical appraisal*" OR "critical assess*" OR "methodolog* apprais*" OR "methodolog* assess*" OR "method* checklist*" OR "methodological rigour" OR "method* quality" OR "credibility assess*" OR "credibility apprais*" OR "credibility checklist*" OR "study apprais*" OR "validity assess*" OR "validity apprais*" OR "validity checklist*") OR ALL=("APPRAISE" OR "AXIS tool" OR "BEES C" OR "BEME" OR "CCAT" OR "CEBMH" OR "CLEAR NPT" OR "Coleman Methodology Score" OR "CSCEDARS" OR "CTAM" OR "Downs and Black" OR "Downs&Black" OR "Downs&Black" OR "Epidemiological Appraisal Instrument" OR "Evidence Project Tool" OR "GRACE" OR "Heyland Methodological Quality Score" OR "Jadad" OR "Jadad Scale" OR "JBI" OR "Maastricht Amsterdam list" OR "MINCIR" OR "MINORS" OR "MMAT" OR "MQRS" OR "NIH quality assessment tool*" OR "NICMAN" OR "NICE tool" OR "Newcastle Ottawa Scale" OR "NOS scale" OR "OHAT" OR "ORBIT" OR "OSQE" OR "Oxford Pain Validity" OR "PEDro" OR "Q Coh" OR "Q Genie" OR "QLADS" OR "QualSyst tool" OR "RCT PQRS" OR "ROB 2" OR "RoB 2.0" OR "ROBINS I" OR "ROBINS E" OR "RoBANS" OR "SIGN" OR "SURE" OR "TESTEX")) AND TS=("target trial*" OR "emulat* trial*" OR "trial emulation*" OR "hypothetical trial*" OR "trial replicat*") | 357 |
| --- | --- | --- |
