# Supplementary material for: Risk-of-Bias Assessment of Target Trial Emulation Studies at Regulatory and Policy Decision Points Along the Medical Product Life Cycle: Protocol for a Scoping Review and Stakeholder Survey
Source: JMIR Res Protoc. 2026 Oct 27;15:e107499. doi: 10.2196/107499 (PMC13752585; doi:10.2196/107499)
Supplement: Supplementary file 4 [file resprot_v15i1e107499_app4.docx]

Table S4 Scoping review – search strategy_ Cochrane Library 13.05.2026 (77 results)

| #6 | #1 OR #2 OR #3 OR #4 in Cochrane Reviews, Cochrane Protocols, Clinical Answers, Editorials, Special Collections | 77 |
| --- | --- | --- |
| #5 | #1 OR #2 OR #3 OR #4 | 1003 |
| #4 | "trial replication" | 77 |
| #3 | "hypothetical trial" | 63 |
| #2 | "emulated trial" OR "trial emulation" OR "emulate a target trial" | 283 |
| #1 | "target trial" OR "target trials" | 825 |
