# Supplementary material for: Risk-of-Bias Assessment of Target Trial Emulation Studies at Regulatory and Policy Decision Points Along the Medical Product Life Cycle: Protocol for a Scoping Review and Stakeholder Survey
Source: JMIR Res Protoc. 2026 Oct 27;15:e107499. doi: 10.2196/107499 (PMC13752585; doi:10.2196/107499)
Supplement: Supplementary file 5 [file resprot_v15i1e107499_app5.docx]

Table S5 Scoping review - search strategy_relevant websites of HTA bodies, regulatory agencies and networks

| **Country** | **Agency/network** | **website** |
| --- | --- | --- |
| Austria | Austrian Federal Office for Safety in Health Care, BASG | <https://www.basg.gv.at/en/> |
|  | Austrian Institute for Health Technology Assessment, AIHTA | <https://aihta.at/page/homepage/en> |
| Belgia | Belgian Healthcare Knowledge Centre, KCE | <https://www.famhp.be/en> |
|  | Federal agency for medicines and health products, FAMHP | <https://www.kce.fgov.be/en> |
| Bulgaria | Bulgarian Drug Agency | <https://bda.bg/en/> |
|  | The National Council on Prices and Reimbursement of Medicinal Products, NCPRMP | [https://www.ncpr.bg/en/#](https://www.ncpr.bg/en/) |
| Canada | Canadian Centre for Applied Research in Cancer Control (ARCC) | <https://cc-arcc.ca/> |
| Croatia | Agency for medicinal products and medical devices of Croatia | <https://www.halmed.hr/en/> |
| Cyprus | Ministry of Health - Pharmaceutical Services | <https://www.moh.gov.cy/moh/phs/phs.nsf/home_en/home_en?openform> |
| Czech | State Institute for Drug Control (National medicines agency) | <https://sukl.gov.cz/en/> |
| Denmark | Danish Medicines Agency | <https://www.laegemiddelstyrelsen.dk> |
|  | Danish Medicines Council | <https://medicinraadet.dk/> |
| Estonia | State Agency of Medicines | <https://www.ravimiamet.ee/en> |
| Europe | European Comission, EC | <https://commission.europa.eu/index_en> |
|  | European Medicines Agency, EMA | <https://www.ema.europa.eu> |
|  | European network for Health Technology Assessment, EUnetHT | <https://tools.eunethta.be/guidelines.html> |
|  | European Network of Centres for Pharmacoepidemiology and Pharmacovigilance, ENCePP | <https://encepp.europa.eu/index_en> |
| Finland | Council for Choices in Health Care in Finland (COHERE Finland) | <https://palveluvalikoima.fi/> |
|  | Finnish Coordinating Center for HTA | <https://fincchta.fi/> |
|  | Finnish Medicines Agency, FIMEA | <https://www.fimea.fi> |
| France | French National Agency for Medicines and Health Products Safety, ANSM | <https://ansm.sante.fr/> |
|  | French National Authority for Health, HAS (Haute Autorité de santé) | https://www.has-sante.fr/jcms/pprd_2986129/en/home |
| Germany | Federal Institute for Drugs and Medical Devices | https://www.bfarm.de/EN/Home/_node.html |
|  | Federal Joint Committee, G-BA | https://www.g-ba.de/english/ |
|  | Institute for Quality and Efficiency in Health Care, IQWiG | <https://www.iqwig.de/en> |
|  | Paul Ehrlich Institute, Federal Institute for Vaccines and Biomedicines | https://www.pei.de/EN/home/home-node.html |
| Global | International HTA database | https://database.inahta.org/ |
|  | Campbell Collaboration | <https://www.campbellcollaboration.org/> |
|  | Cochrane Bias Methods Group, BMG | <https://methods.cochrane.org/bias/> |
|  | Enhancing the QUAlity and Transparency Of health Research network, Equator | <https://www.equator-network.org/> |
|  | International Society for Pharmacoepidemiology, ISPE | <https://www.pharmacoepi.org/> |
|  | The Professional Society for Health Economics and Outcomes Research, ISPOR | <https://www.ispor.org/home> |
|  | Joanna Briggs Institute, JBI | <https://jbi.global/> |
|  | RWE4Decisions | <https://rwe4decisions.com/> |
| Greece | National Organization for Medicines | https://www.eof.gr/en/ |
| Hungary | National Centre for Public Health and Pharmacy | https://nnk.gov.hu/ |
| Iceland | Icelandic Medicines Agency | https://www.ima.is/ |
| Ireland | Health Information and Quality Authority, HIQA | https://www.hiqa.ie/ |
|  | Health Products Regulatory Authority, HPRA | https://www.hpra.ie/ |
|  | The National Centre for Pharmacoeconomics | https://www.ncpe.ie/ |
| Italy | Italian Medicines Agency | https://www.aifa.gov.it/en/home |
| Italy | The National Agency for Regional Health Services, AGENAS | https://www.agenas.gov.it/ |
| Latvia | The State Agency of Medicines of Latvia | https://www.zva.gov.lv/en |
| Liechtenstein | Liechtenstein Office of Health / Department of Pharmaceuticals | https://www.llv.li/en |
| Lithuanina | State Medicines Control Agency of Lithuania | https://vvkt.lrv.lt/en/ |
| Luxembourg | Ministry of Health and Social Security | https://santesecu.public.lu/en.html |
| Malta | Malta Medicines Authority, MMA | https://medicinesauthority.gov.mt/ |
| Netherlands | Central Committee on Research Involving Human Subjects | https://english.ccmo.nl/ |
|  | Health and Youth Care Inspectorate, Ministry of Health, Welfare and Sport | https://english.igj.nl/ |
|  | Medicines Evaluation Board | https://www.cbg-meb.nl/ |
|  | National Health Care Institute, Zorginstituut Nederland, ZIN | https://english.zorginstituutnederland.nl/ |
| Norway | Norwegian Medical Products Agency | https://www.dmp.no/en |
|  | Nye Metoder | https://www.nyemetoder.no/en/english/ |
|  | The Norwegian Institute of Public Health, NIPH | https://www.fhi.no/en |
| Poland | Agency for Health Technology Assessment and Tariff System, AOTMiT | https://www.aotm.gov.pl/en/ |
|  | Chief Pharmaceutical Inspectorate | https://archiwum.gif.gov.pl/en |
|  | Office for Registration of Medicinal Products, Medical Devices and Biocidal Products | https://www.gov.pl/web/urpl/ |
| Portugal | National Authority of Medicines and Health Products, INFARMED | https://www.infarmed.pt/web/infarmed-en/ |
| Romania | National Agency for Medicines and Medical Devices of Romania | https://www.anm.ro/en/ |
| Slovakia | Agency for Medicinal Products and Medical Devices of the Republic of Slovenia | https://www.jazmp.si/en/ |
|  | National Institute for Value and Technologies in Healthcare, NIHO | https://niho.sk/en/ |
|  | State Institute for Drug Control | <https://www.sukl.sk/> and <https://povodny.sukl.sk/> |
| Spain | Spanish Agency Of Medicines And Medical Devices | <https://www.aemps.gob.es/> |
| Sweden | Swedish Agency for Health Technology Assessment and Assessment of Social Services, SBU | <https://www.sbu.se/en/> |
|  | Swedish Medical Products Agency | <https://www.lakemedelsverket.se/en> |
| United Kingdom | Medicines & Healthcare products Regulatory Agency, MHRA | <https://www.gov.uk/government/organisations/medicines-and-healthcare-products-regulatory-agency> |
|  | National Institute for Health and Care Excellence, NICE | <https://www.nice.org.uk> |
|  | National Institute for Health and Care Research, NIHR | <https://www.nihr.ac.uk/> |
| United States | Agency for Healthcare Research and Policy, AHRQ | <https://www.ahrq.gov/cpi/index.html> |
|  | Food and drug administration, FDA | <https://www.fda.gov/> |
